# Impact of truncal vagotomy on complicated peptic ulcer after distal gastrectomy with reconstruction by jejunal pouch interposition

**DOI:** 10.1186/s40792-020-00879-w

**Published:** 2020-06-01

**Authors:** Reika Yamashita, Naoto Takahashi, Kazuto Tsuboi, Norio Mitsumori, Hideyuki Kashiwagi, Katsuhiko Yanaga

**Affiliations:** 1 Department of Surgery, The Jikei University Kashiwa Hospital, 163-1 Kashiwa-shita, Kashiwa, Chiba Prefecture 277-8567 Japan; 2 Department of Surgery, The Jikei University School of Medicine, 3-19-18 Nishi-shinbashi, Minato-ku, Tokyo, 105-8471 Japan; 3 Department of Surgery, Fuji City General Hospital, Fuji, Shizuoka Prefecture Japan

**Keywords:** Truncal vagotomy, Peptic ulcer, Pouch interposition, Distal gastrectomy, Jejunal pouch

## Abstract

**Background:**

We encountered a case of marginal ulcer in the jejunum after distal gastrectomy with jejunal pouch interposition. However, it has not been reported and not confirmed the treatment. We chose truncal vagotomy, considering reduced morbidity and postoperative complications.

**Case presentation:**

A case was a 69-year-old woman who was admitted to our hospital with melena. She had received curative distal gastrectomy with a 15-cm jejunal pouch reconstruction for early gastric cancer. Marginal ulcer in the jejunal pouch was detected by upper gastrointestinal endoscopy. She was given medication; however, she repeated hospitalization for melena and abdominal pain. Therefore, we decided to perform surgery, and truncal vagotomy was performed. The patient’s postoperative course was uneventful and was discharged on the 22nd postoperative day. Symptoms such as abdominal pain and melena were improved after truncal vagotomy.

**Conclusion:**

We presented a case with a complicated peptic ulcer after distal gastrectomy with reconstruction by jejunal pouch interposition, which was successfully treated by truncal vagotomy, a surgical acid-reducing procedure which does not require resection of remnant stomach.

## Background

Over the last half-century, the epidemiology of both gastric and duodenal ulcer disease has changed markedly [1]. The discovery of antisecretory agents, as well as recognition of the role of Helicobacter pylori in ulcer pathophysiology, has led to the effective non-operative treatment of peptic ulcer disease [2, 3]. Consequently, the elective gastric and duodenal ulcer surgeries have replaced by complications of peptic ulcer disease, including perforation and bleeding, which require emergency surgery.

Recently, because of the increasing number of early gastric cancer and improvements in its survival rates, greater attention has been directed towards the quality of life and the nutritional status of patients with gastric cancer after surgery. However, conventional reconstructions, namely Billroth I, Billroth II, and Roux-en-Y are known to exhibit certain limitations, such as a small reservoir and food stasis. Also, all known procedures are inevitably accompanied by postgastrectomy syndrome, which may involve weight loss caused by eating restrictions, heartburn, loss of appetite, and changes in daily activity, all of which are consistent with a decline in physical strength. Jejunal pouch interposition between the remnant stomach and duodenum has been introduced for conventionally open distal gastrectomy, not only to substitute for the small reservoir but also to maintain a physiologic pathway for ingested foods and to alleviate postgastrectomy syndrome. The usefulness of jejunal interposition for gastric substitution has since been reported by several authors [4–9]. However, complications of the interposition have not been elucidated.

We encountered a case of marginal ulcer in the jejunum after distal gastrectomy with jejunal pouch interposition. However, to our knowledge, complications of this reconstruction, especially the development of an ulcer in the jejunal pouch, have not been reported. Although several surgical options such as conversion from jejunal pouch interposition to Roux-en-Y or Billroth I reconstruction were present, we underwent truncal vagotomy for reducing morbidity and postoperative complications [10–12].

## Case presentation

**Fig. 1:**
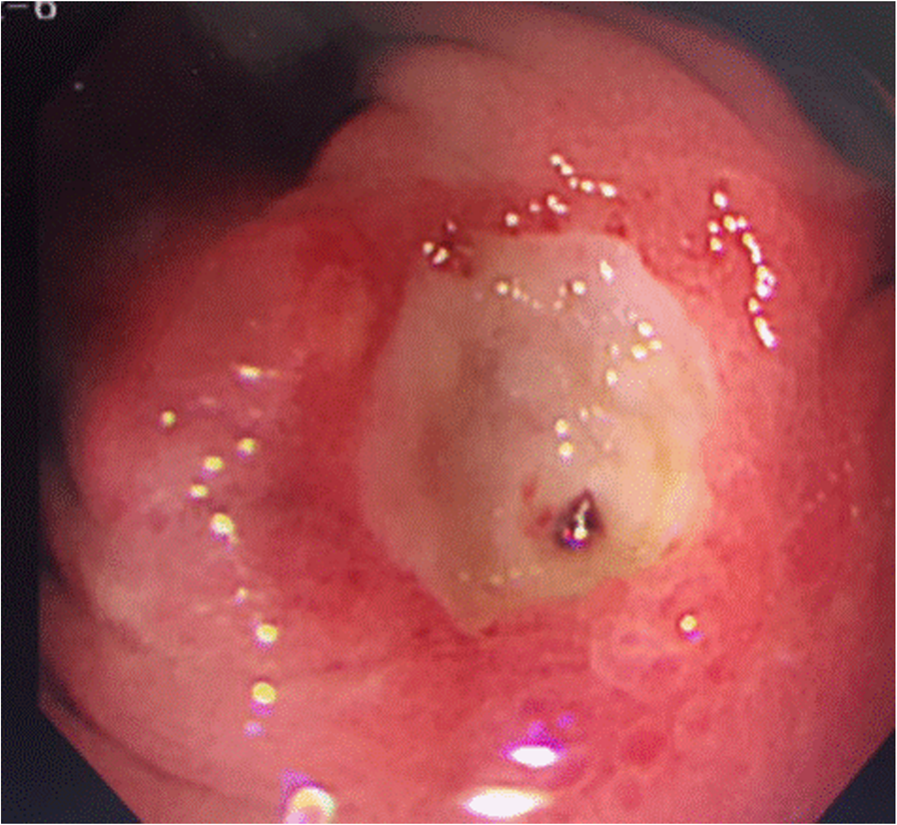
Marginal ulcer in the jejunal pouch was detected by upper gastrointestinal endoscopy

**Fig. 2:**
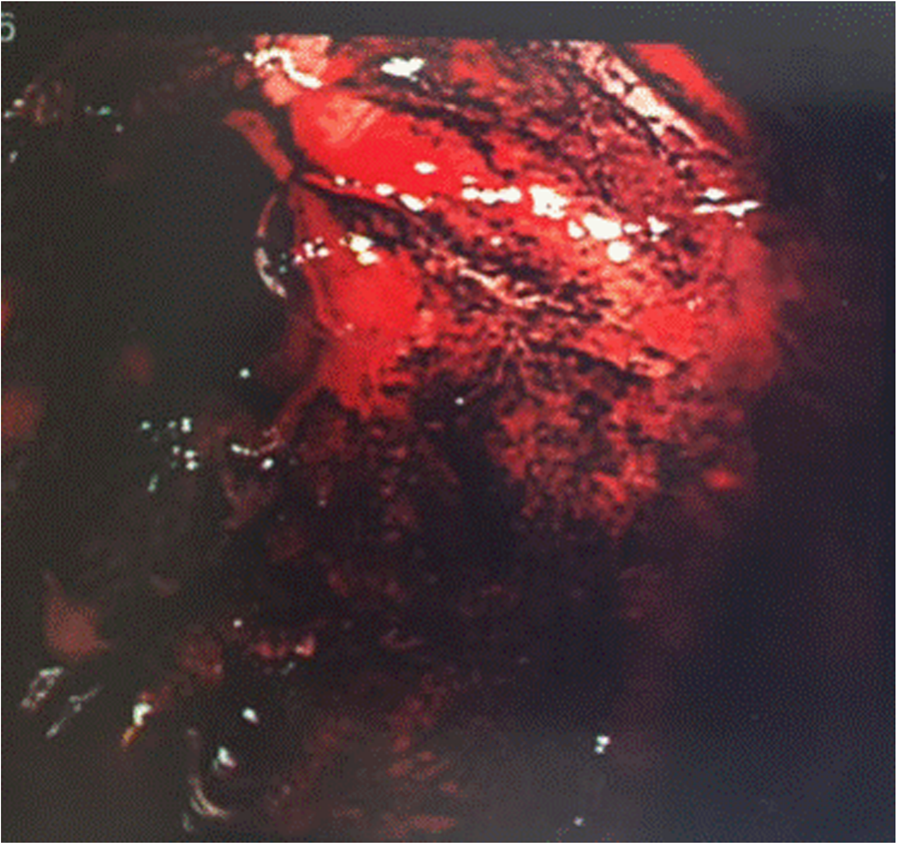
Any area of the mucosa which turned black-blue (pH < 3.0) within the first 3 min

A 69-year-old woman was admitted to our hospital with melena, who received curative open distal gastrectomy with a 15-cm jejunal pouch reconstruction for early gastric cancer 5 years ago. Histopathological results showed that the depth of cancer was within the mucosal layer with the peptic ulcer and no lymph node metastasis of forty-two resected lymph nodes. Marginal ulcer in the jejunal pouch was detected by upper gastrointestinal endoscopy (Fig. 1), for which endoscopic clipping was performed. The patient was given a proton pump inhibitor (PPI) and was managed as an outpatient. However, due to melena and abdominal pain, repeated hospitalization was required. Endoscopic Congo Red test was performed according to Donahue et al. [13] under basal conditions without gastric acid stimulation. After aspiration of all gastric contents, a solution of 0.5% Congo red in 5% bicarbonate was sprayed through the endoscope to the gastric mucosa. Any area of the mucosa which turned black-blue (pH < 3.0) within the first 3 min was considered positive (Fig. 2). Simultaneous ambulatory 24-h multichannel impedance-pH monitoring [14] data showed that the remnant stomach had the secretion of gastric acid (Table 1), which was judged to be responsible for the peptic ulcer in the jejunal pouch.

**Table 1:** pH monitoring

	Pre-operative	Post-operative
Fraction time pH below 4 (%)	61.8	5.9

**Fig. 3:**
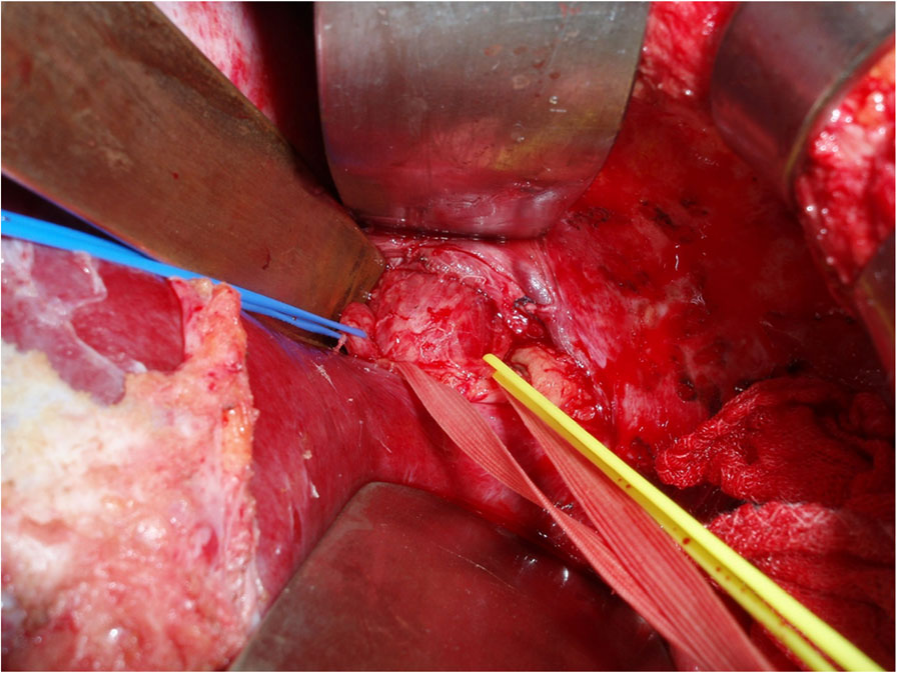
The abdominal esophagus and esophagogastric junction

Truncal vagotomy was performed as follows: upper-middle laparotomy was performed with severe adhesion between the abdominal wall and upper abdominal organs. The lateral segment of the liver was retracted cranially, and the subphrenic abdominal esophagus was appeared with care, not to injure the liver and the remnant stomach. The abdominal esophagus was gradually exposed, and EGJ (esophagogastric junction) was pulled vertically and caudally by a tape to separate the anterior and posterior vagal trunk (Fig. 3). Both vagal trunks were resected for 1 cm in length in order to cut the nerve fibers completely.

The patient’s postoperative course was uneventful and was discharged on the 22nd postoperative day. A 24-h pH monitoring after surgery demonstrated an improvement of the acidic phase in the remnant stomach (Table 1). Symptoms of abdominal pain and melena were improved after truncal vagotomy.

## Discussion

The most popular reconstruction method of distal gastrectomy has been either Billroth I or Roux-en-Y [15]. However, the postoperative course of these reconstruction methods has been unfavorable, due to problems such as postgastrectomy disorder, microgastria, early dumping syndrome, reflux esophagitis, alkaline gastritis of the remnant stomach, and postprandial stasis of the remaining stomach. Jejunal pouch interposition between the remnant stomach and duodenum has recently been introduced for distal gastrectomy, not only to substitute for the small reservoir but also to maintain a physiologic pathway for ingested foods and to alleviate postgastrectomy syndrome [4–9]. There have been no studies with long-term follow-up after jejunal pouch interposition, and to the best of our knowledge, the development of an ulcer in the jejunal pouch after distal gastrectomy has not been reported.

In this case, vagus nerve-preserving distal gastrectomy with the reconstruction of jejunal pouch interposition was performed. Marginal ulcers are relatively rare complications after gastrectomy if the truncal vagotomy is incorporated [15]. In distal gastrectomy for early gastric cancer, truncal vagotomy is not essential for dissection of the cardiac lymph nodes, and recently, preservation of the vagal nerves has been performed to prevent postoperative disorders such as postprandial stasis. Thus, when the fundic gland area is left in the stomach after the vagus nerve-preserving gastrectomy, the acid secretion function of the stomach is mostly preserved. In the current case, Congo Red test and 24-h pH monitoring examination showed that the remnant stomach contained secretions of gastric acid (Fig. 2 and Table 1).

Elective surgery for peptic ulcer disease has recently become obscured from the medical world because of the significant advances in medical treatment over the past three decades [16–18]. This technique is now preserved only for patients with complications of peptic ulcer disease intractable bleeding or gastroduodenal perforation. However, the current case offers a view that truncal vagotomy is still an essential technique to control bleeding from peptic ulcer after gastrectomy. There are several reasons why truncal vagotomy is still an essential technique. First of all, although this technique is outdated [19], it still can be used in cases when the Billroth I and the Roux-en-Y fails or is not an option. Furthermore, unlike the Billroth I and the Roux-en-Y techniques, truncal vagotomy is free of the risk of complications stemming from leakage from the reconstructed area. This is because truncal vagotomy decreasing the gastric acid functions within the remaining stomach does not require re-reconstruction using the duodenum or the jejunum.

## Conclusions

We presented a case with a complicated peptic ulcer after distal gastrectomy with reconstruction by jejunal pouch interposition, which was successfully treated by truncal vagotomy, a surgical acid-reducing procedure which does not require resections.

## Data Availability

All data generated or analyzed during this study are included in this published article.

## Abbreviation

PPI: Proton pump inhibitor
